# A Chip Off the Old Block? The Relationship of Family Factors and Young Adults’ Views on Aging

**DOI:** 10.3389/fpsyg.2022.808386

**Published:** 2022-02-18

**Authors:** Cathy Hoffmann, Anna E. Kornadt

**Affiliations:** Department of Behavioral and Cognitive Sciences, University of Luxembourg, Esch-sur-Alzette, Luxembourg

**Keywords:** aging, stereotypes, family, views on aging, self-perceptions of aging, intergenerational contact

## Abstract

Views on aging (VoA), such as self-perceptions of aging or age stereotypes are generated in early childhood and continue to develop throughout the entire lifespan. The ideas a person has about their own aging and aging in general influence their behavior toward older persons as well as their own actual aging, which is why VoA are already important in adolescence and young adulthood. The current study investigates VoA of young adults in different domains (continued growth, physical decline, social loss) and how different family aspects are related to VoA. From February to March 2021, *N* = 305 young adults [aged 18–30 years, *M*_age_(SD) = 22.20 (2.60)] participated in an online survey, in which, in addition to sociodemographic variables and family aspects (contact with grandparents, family age climate, i.e., the frequency and valence of talking about age in the family), self-perceptions of aging, age stereotypes, and the young adults’ ratings of their parents’ VoA were assessed. The results of stepwise regression analyses predicting the young adults’ VoA, revealed significant associations between the quality of contact with grandparents and the self-perceptions of aging of young adults. However, the frequency of contact was neither related to young adults’ self-perceptions of aging nor age stereotypes. Grandparents’ health status emerged as a significant moderator between the relationship of contact quality and the young adults’ self-perceptions of aging as continued growth and physical decline. Family climate was also found to be significantly related to young adults’ self-perceptions of aging. Similarities regarding VoA within the family were demonstrated, based on proxy report from the respondents. The results underline the importance of family aspects for the development of VoA in young adulthood, and the significance of interventions targeting these factors to combat ageism.

## Introduction

Views on aging (VoA) is an umbrella term, encompassing constructs that represent individuals’ conceptions about the process of aging, older people, and old age in general (age stereotypes) as well as ones’ own aging (self-perceptions of aging; Wurm et al., 2017). VoA are already generated in early childhood and continue to develop throughout the entire life span (Levy, 2009), however, these views also seem to be considerably stable throughout life (Wurm and Huxhold, 2012). Stereotypes that are endorsed and internalized early in life turn into aging self-stereotypes as people get older, a process that is described in Stereotype Embodiment Theory (SET, Levy, 2009). In general, it is assumed that both, self-perceptions of aging and age stereotypes, inform future expectations of the self (Ryff, 1991; Rothermund and Brandtstädter, 2003) as well as ones’ own behavioral choices and later life goals (Kornadt and Rothermund, 2012; Lloyd et al., 2018). Furthermore, negative VoA also influence later life outcomes via behavioral (e.g., decreased engagement in health behaviors) and physiological (e.g., increased stress responses) pathways (Levy, 2009). Thus, it is of great importance to investigate how VoA are formed and characterized in younger years (e.g., Kornadt et al., 2020).

A growing body of research has already provided evidence that VoA endorsed in earlier life have an influence on behavior toward older people, for example leading to ageism in terms of overaccommodating speech, avoidance, and other discriminatory actions (Mahne et al., 2017; Flamion et al., 2020a; Lapp et al., 2020). In addition, as people move through the life span and get older themselves, VoA also affect a person’s own health outcomes and wellbeing. Numerous studies have shown that negative VoA in earlier life are related to poorer health outcomes in later life (Levy et al., 2002, 2009; Blawert et al., 2020). This suggests that young adults who associate aging for example with physical decline might experience a negative impact on their own health in later life (e.g., Lloyd et al., 2018).

Based on these findings that young adults’ VoA affect their behavior toward older people and that VoA also represent the basis for self-stereotypes of aging as people get older themselves, it is of utmost importance to understand the development of VoA in young people (Kornadt et al., 2020). So far, empirical studies have mostly focused on the existence and characteristics of VoA in childhood, in adolescence and in young adulthood (Bergman, 2017; Flamion et al., 2020b) and on intergenerational contact as a relevant factor in the development of VoA. However, we still know relatively little about the family as a context for the developmental process of VoA in younger years (Kornadt et al., 2020). The current study thus set out to investigate how contact with grandparents, family age climate (i.e., the frequency and valence of talking about age in the family) and parents’ VoA are related to young adults’ VoA.

### Intergenerational Contact—The Role of Grandparents

As described in intergroup contact theory (Allport et al., 1954), greater contact is related to less prejudice toward a specific stigmatized group. This also seems to be the case for older adults. In a systematic review, Marques et al. (2020) found that contact with older people (especially contact of high or positive quality) seems to reduce the prevalence of ageism and represents one factor robustly associated with ageism (or lack thereof). In that regard, the relationship between children and their grandparents seems to play a special role in their development of VoA, as grandparents are usually the first older people that children meet as well as being those that are most visible and important (Christian et al., 2014). A number of studies have demonstrated that if intergenerational contact is present within the family, children are more likely to have positive VoA (e.g., Gilbert and Ricketts, 2008; Attar-Schwartz et al., 2009). However, there are still many questions regarding whether only quality of contact with grandparents is related to VoA or if frequency of contact is also a factor (see Flamion et al., 2019 for an overview). In addition, many studies have investigated the role of contact in childhood and adolescence while there is relatively little research focusing on young adults.

In addition, besides mere contact, grandparents’ health might represent another essential factor relevant for young adults’ VoA (Flamion et al., 2019; Blawert et al., 2020). In a study by Flamion et al. (2019), for example, grandparents’ health status turned out to be an important factor besides the role of contact: Children and adolescents aged 7–16 years who described their grandparents’ health as very good had significantly more positive VoA compared to participants who rated their grandparents’ health as only good or not so good. In addition to this main effect, the health status of a grandparent might also moderate the impact of contact: Since declining health is one of the most pervasive, negative stereotypes of old age (e.g., Kornadt et al., 2018), having it confirmed in a grandparent might override the positive effects of contact. Thus, if a grandparent is sick, this might taint young adults’ VoA in the health domain, even if they experience good contact with them. Taken together, as intergenerational contact outside the family is rare (e.g., Drury et al., 2016), grandparents might often not only be the first and most significant contact that young adults have with older people, but also the only older people that they have contact with. For this reason, their status and characteristics are of special importance for the formation of VoA. It is therefore important to understand which characteristics and contextual factors of the child-grandparent relationship are important for VoA development.

### Family as a Context of Views on Aging Development

Contact with grandparents seems to be of great significance, but other family characteristics can be important for VoA development as well. In general, the family serves as a basis for value and attitude development in children. As the first instance of socialization, knowledge is shared by parents and siblings, family practices embody values and attitudes and social learning processes work via interaction and observation (Gilbert and Ricketts, 2008; Albert and Ferring, 2012; Mendonça et al., 2018). It has been shown in a number of contexts that attitudes and values are passed on within the family and that there are similarities in parents’ and children’s values and attitudes (Schönpflug, 2008; Schönpflug and Bilz, 2009; Albert and Ferring, 2012). In a study by Schönpflug and Bilz (2009) for example, an authoritative parenting style characterized by warmth and responsiveness turned out to be an effective transmission process for various values and beliefs between parents and their children. Other constructs, such as gender roles, attitudes toward risk and race attitudes are also intergenerationally transmitted (Gauly, 2017; Bredtmann et al., 2020; Wolff, 2020). For instance, Chen and Chang (2017) investigated whether socialization in the primary family is a significant predictor of children’s gender role attitudes. Results showed that parents had strong effects on the development of children’s gender role attitudes and that the children’s gender role attitudes mirrored their social background. These findings provide evidence for the intergenerational transmission of different kinds of values and attitudes.

However, despite acknowledging the importance of the family for VoA formation (Gilbert and Ricketts, 2008) research on the role of the family for VoA development has so far been limited. In SET (Levy, 2009), the importance of early age stereotype socialization and internalization is explicitly mentioned. How age is dealt with in the family, for example via modeled behavior toward older adults, via old age portrayal in media consumed by parents and children alike, and how age is discussed in the family, provides context for how children see older people and old age. Despite this, empirical research on family factors affecting VoA is rather scarce. In a study of children aged 6–14 years, Lineweaver et al. (2017) found similarities between children’s age stereotypes and those of their parents, and this relationship was stronger for older children, pointing to possible cumulative effects of social learning and cognitive development. Kennison and Byrd-Craven (2020) found that a strained mother-child relationship and avoidant attachment style in childhood predicted ageism in young adulthood. For older adults, Kim et al. (2021) found that parents’ aging conditions (financial, health-related) influenced their grown-up children’s aging anxiety. However, to the best of our knowledge, no study has thus far investigated to what extent family age climate and parents’ VoA are related to young adults’ age stereotypes and self-perceptions of aging.

### Aims and Hypotheses

The current study extends previous literature by focusing on the question of how different family factors are related to young adults’ VoA. We further extend previous studies by taking the multidimensionality of VoA into account and investigating young adults’ self-perceptions of aging as well as their age stereotypes within different dimensions (continued growth, physical decline, and social loss). VoA are inherently multidimensional (Kornadt et al., 2020), and children already have differentiated views of older people that take into account different functional domains, as well as differences between their own aging and older people in general (e.g., Lloyd et al., 2018; Flamion et al., 2019).

Referring to studies on intergenerational contact and the important role of grandparents in younger years, we hypothesized that contact between young adults and their grandparents would be related to young adults’ VoA by assuming that both contact frequency (H1) and contact quality (H2) are positively related to VoA. Further, we predicted that grandparents’ health status would moderate the relationship between contact quality and VoA of young adults. The better the contact with grandparents, the more positive/less negative young adults’ VoA, but only if the grandparents are in good health (H3). Taking into account theories of stereotype matching (Levy and Leifheit-Limson, 2009), this should be especially the case for VoA as physical decline.

Furthermore, taking family value transmission and social learning processes into account, we assumed that there would be similarities in parents’ and young adults’ VoA, i.e., the more positive/negative parents’ VoA, the more positive/negative young adults’ VoA, respectively (H4) and that family age climate and parents’ VoA are related to young adults’ VoA. We hypothesized that an appreciative family climate in the primary family on aging would be related to more positive/less negative VoA (H5).

To investigate the robustness of these effects, we explored whether the relationship between variables remained stable when including young adults’ health status, age and gender. These variables are usually used as controls in VoA research. Because of its relevance for the current research question, we also controlled for contact with older people outside the family. For analyses regarding family age climate and the relationship between parents’ and children’s VoA, we additionally controlled for parents’ age.

## Materials and Methods

### Sample and Procedure

*N* = 305 participants, aged 18–30 years (*M*_age_ = 22.20, *SD* = 2.60) from the Grand-Duchy of Luxembourg were recruited to participate in an online survey. 73.8% of the sample were female, 85.9% had Luxemburgish nationality, 59% were students and the majority were still living with their parents (64.6%). Further relevant sociodemographic information is presented in Table 1. The questionnaire was created using the platform SoSci Survey and shared via social networks such as Facebook and Instagram for a duration of 1 month, from February 25th to March 25th, 2021. Participants answered questions concerning sociodemographic information, self-perceptions of aging, age stereotypes as well as the perceived VoA of their parents. Other family aspects related to age were also assessed in the questionnaire such as the quantity and quality of contact with grandparents, the grandparents’ health status and the family climate in the primary family regarding aging and older people. Completing the questionnaire took 20–25 min and the survey could be paused and returned to at any time. The language of the survey was German. Psychology students of the University of Luxembourg received 25 min of participation credit. No other incentives were offered. The study received Ethics Approval as foreseen by the Ethics Review Panel of the University of Luxembourg for final year theses. A total of *N* = 497 people started the questionnaire, of which *N* = 192 (38.6%) dropped out.

**TABLE 1:** Sociodemographic information for the total sample.

	Total sample (*N* = 305)
**Sex**	
Female, *n* (%)	225 (73.8)
Male, *n* (%)	79 (25.9)
Other or n/a	1 (0.3)
Age, *M* (*SD*)	22.20 (2.60)
**Marital status, *n* (%)**	
Single	190 (62.3)
Married	4 (1.3)
Single, in relationship	97 (31.8)
In a registered relationship	11 (3.6)
Widowed	2 (0.7)
Other or n/a	1 (0.3)
**Current Activity/Employment, *n* (%)**	
High school student	44 (14.4)
University student	180 (59.0)
Employed	70 (23.0)
Unemployed	5 (1.6)
Other or n/a	6 (2.0)
**Living situation**	
With my parents	197 (64.6)
Alone	22 (7.2)
With my partner	42 (13.8)
In a shared flat	38 (12.5)
Other or n/a	6 (1.9)
**Nationality**	
Luxembourgish	262 (85.9)
German	25 (8.2)
French	1 (0.3)
Portuguese	9 (3.0)
Other or n/a	8 (2.6)
**Educational background**	
Primary school	26 (8.5)
High school degree	112 (36.7)
Secondary school degree	49 (16.1)
Technician’s diploma	6 (2.0)
Apprenticeship or vocational school	9 (3.0)
Bachelor	69 (22.6)
Master	12 (3.9)
Other or n/a	22 (7.2)

### Measures

#### Personal Information

Information on participants’ socio-demographic characteristics included gender, age, educational status, marital status as well as their current living situation. Table 1 shows the categories that were used to assess sociodemographic information. We also assessed participants’ subjective health by asking them how they rated their general state of health (1 = very bad to 5 = very good). Additionally, the parents’ and grandparents’ sociodemographic information was assessed, such as their age, information regarding the parents’ educational level and professional situation as well as the current living situation of the grandparents. Participants indicated whether their grandparents were still living at home or somewhere else, for example a nursing home, an assisted living facility or a multigenerational house.

#### Views on Aging

Young adults’ self-perceptions of aging, age stereotypes and the parents’ VoA were assessed using the AgeCog scale (Steverink et al., 2001). The scale, consisting of a total of 12 items (four per domain), was designed to capture the VoA in the domains of Continued growth (CG, sample item “Aging means to me that I continue to realize my ideas”), “Social loss (SL, e.g., ‘Aging means to me that I feel lonely more often’)” and “Physical decline (PD, e.g., ‘Aging means to me that I am less vital and fit.’).” For age stereotypes, the introductory sentence started with “Aging means to most other people that….” To assess estimations of the parents’ self-perceptions of aging, the same items were introduced again at the end of the questionnaire, this time with the instruction to “Aging means to my mother/father….” Participants had to rate all items on a four-point Likert scale from “completely applies” to “does not apply at all.” All items were reverse coded so that higher scores indicated more endorsement and sum scores were computed for each scale from the respective items.

#### Grandparents

Contact frequency with grandparents was assessed by asking “Overall, how often do you have contact with your grandparents?” (1 = almost never to 5 = very often). Contact quality was assessed by asking: “Overall, how would you rate the quality of contact with your grandparents?” (1 = very poor to 5 = very good). For grandparents’ health status, participants had to indicate “Overall, how would you rate your grandparents’ state of health? (1 = very poor to 5 = very good). Participants were instructed to answer these questions by thinking of their grandparents in general. If there was a large difference between the grandparents, participants should refer to the grandparent with whom they had the most and the best contact and whose health was best.

#### Family Age Climate

We assessed the frequency and valence of age-related conversations within the primary family. Participants were asked to indicate on a four-point Likert scale how often the topic of aging and being old was discussed in the primary family (1 = never to 4 = frequently). The valence of family climate, rated on a five-point Likert scale, intended to provide information about the quality of the family climate toward aging “If there is conversation about aging and being old in your family, is this rather 1 = negative to 5 = positive”? *N* = 29 subjects stated that the topic of aging was never discussed in their primary family and were therefore excluded from the subsequent analysis regarding the valence of family age climate.

#### Contact

Young adults’ contact with older people outside the family was assessed (“Do you have contact with older people outside your family?” 1 = yes/2 = no) and if they answered in the affirmative, also the quality of this contact (“Overall, how would you rate the quality of this contact,” 1 = very poor to 5 = very good). Most participants (*n* = 265, 87%) indicated having no contact with older people outside the family.

### Analyses

SPSS 27 was used for all analyses and data as well as syntax for all analyses are accessible at https://osf.io/z74vf/?view_only=090e504449c7435b8769db6721ccbe14. First, descriptive statistics and correlations were computed to address means and bivariate relationships between the variables. To test our hypotheses and to estimate the incremental contribution of the respective predictors and covariates, stepwise regression analyses were calculated. First, the respective predictor variables were included in the regression models. For grandparents, first frequency of contact was added, then quality of contact. To address the impact of the grandparents’ health status on the relationship between contact quality and VoA, predictors were standardized before analyses and grandparents’ health status was included as a predictor, as well as the interaction term between quality of contact and health status (Hayes, 2018). For family factors, first the parents’ VoA were included, followed by the frequency and valence of family conversations regarding age. In a final step for both analyses, control variables were included to test the robustness of associations in the presence of these covariates: Participants’ age, gender (1 = male, 2 = female), and health status were included as is common practice in studies on VoA; due to their potential relevance for the current research question, contact with older people outside the family (1 = yes, 2 = no), and for analyses on family climate also parents’ ages were added as additional covariates.

## Results

Descriptive statistics and bivariate correlations for all variables are presented in Table 2.

**TABLE 2:** Descriptive statistics for all study variables and bivariate correlations for young adults’ VoA with all study variables.

Variable	*n*	*M*	*SD*	CG_SPA	PD_SPA	SL_SPA	CG_AS	PD_AS	SL_AS	α
Continued growth (CG_SPA)	305	2.99	0.56	−						0.78
Physical decline (PD_SPA)	305	2.79	0.55	−0.49%**	−					0.77
Social loss (SL_SPA)	305	2.04	0.55	−0.56%**	0.52%**	−				0.70
Continued growth (CG_AS)	303	2.40	0.61	0.36%**	−0.33%**	−0.30%**	−			0.83
Physical decline (PD_AS)	303	3.22	0.55	−0.15%**	0.50%**	0.20%**	−0.55%**	−		0.74
Social loss (SL_AS)	303	2.60	0.59	−0.23%**	0.31%**	0.41%**	−0.66%**	−0.62%**	−	0.82
Continued growth (CG_Parents)	300	2.84	0.53	0.40%**	−0.32%**	−0.28%**	0.23%**	−0.21%**	−0.22%**	0.86
Physical decline (PD_Parents)	300	2.95	0.52	−0.25%**	0.48%**	0.20%**	−0.17%**	0.11%**	0.37%**	0.83
Social loss (SL_Parents)	300	2.15	0.57	−0.36%**	0.27%**	0.48%**	−0.22%**	0.42%**	0.17%**	0.75
Contact frequency	293	3.27	1.18	–0.03	0.04	–0.07	–0.04	0.03	0.01	
Contact quality	290	4.15	1.00	0.10	−0.15%*	−0.17%**	–0.04	–0.07	–0.09	
Family age climate (frequency)	305	2.78	0.85	–0.01	0.03	0.10	–0.04	0.08	0.01	
Family age climate (valence)	279	2.80	0.79	0.25%**	0.36%**	−0.27%**	0.16%**	−0.17%**	–0.07	
Age	305	22.20	2.60	0.07	0.00	–0.04	–0.05	0.01	0.02	
Age father	299	54.35	6.68	–0.04	0.00	–0.02	–0.06	0.02	0.01	
Age mother	304	52.48	4.81	0.04	0.09	0.00	–0.05	0.11	0.08	
Health status of grandparents	289	3.46	0.93	0.00	–0.06	–0.02	–0.05	0.04	0.05	
Personal health status	305	4.29	0.76	0.20%*	−0.15%*	−0.23%**	0.08	–0.03	–0.11	
Contact with older people outside the family (1 = yes, 2 = no)	305			–0.03	0.03	0.00	–0.11	0.05	0.07	
Gender (1 = male, 2 = female)	305			–0.04	0.12%*	0.11	–0.02	0.12%*	0.12%*	

SPA, self-perceptions of aging; AS, age stereotypes; *p < 0.05, **p < 0.001.

Self-perceptions of aging as physical decline and social loss were significantly related to all other variables, except for the frequency of contact with grandparents as well as the frequency of talking about age in the family. All three domains of self-perceptions of aging and age stereotypes correlated significantly with all three domains of the parents’ VoA. For self-perceptions of aging and age stereotypes as physical decline there was a significant and high correlation (*r* = 0.50, *p* < 0.001) while for VoA as continued growth and social loss there was a moderate correlation. Age stereotypes as continued growth and physical decline were not significantly related to the variables regarding contact with grandparents and the frequency of talking about age in family, however for the valence of talking about age in the family, a significant and high correlation could be observed. Age stereotypes as social loss was neither related to any variable regarding contact with grandparents, nor to any of the family climate variables (Table 2).

### The Role of Grandparents

To address the relationship of VoA with quality and quantity of contact with grandparents, we ran stepwise multiple regressions with the respective VoA as the outcome variable (see Tables 3, 4). Contrary to hypothesis 1, which stated that more frequent contact should be related to more positive, VoA, frequency of contact with grandparents was not significantly related to any of the three domains of self-perceptions of aging, and neither to age stereotypes (Table 3, Model 1 for self-perceptions of aging; Table 4, Model 1 for age stereotypes). In terms of hypothesis 2, which stated that higher contact quality should be related to more positive VoA, quality of contact as an additional predictor was indeed significantly related to all three domains of self-perceptions of aging (Table 3, Model 2) whereas no significant effect could be observed for the respective age stereotypes (Table 4, Model 2)^1^.

**TABLE 3:** The role of grandparents for young adults’ self-perceptions of aging (SPA).

	Continued growth (CG_SPA)					Physical decline (PD_SPA)					Social loss (SL_SPA)				
Variables	*B*	*SE* (*B*)	β	*R* ^2^	*ΔR* ^2^	*B*	*SE* (*B*)	β	*R* ^2^	*ΔR* ^2^	*B*	*SE (B)*	β	*R* ^2^	*ΔR* ^2^
Model 1				0.00	0.00				0.00	0.00				0.01	0.01
Constant	2.98	0.03				2.8	0.03				2.05	0.03			
Frequency of contact	–0.02	0.03	–0.03			–0.03	0.03	–0.06			–0.05	0.03	–0.08		
Model 2				0.02*	0.02*				0.02*	0.02*				0.03**	0.02**
Constant	2.98	0.03				2.8	0.03				2.05	0.03			
Frequency of contact	–0.07	0.04	–0.12			0.02	0.04	0.04			0.01	0.04	0.01		
Contact quality	0.10	0.04	0.17*			–0.09	0.04	−0.17*			–0.10	0.04	−0.18**		
Model 3				0.02	0.00				0.02*	0.00				0.03*	0.00
Constant	2.98	0.03				2.8	0.03				2.05	0.03			
Frequency of contact	–0.07	0.04	–0.13			0.03	0.03	0.05			0.01	0.04	0.01		
Contact quality	0.10	0.04	0.17*			–0.09	0.40	−0.17*			–0.10	0.04	−0.18*		
Health status of grandparents	0.01	0.03	0.01			–0.03	0.03	–0.05			0.00	0.03	0.00		
Model 4				0.03	0.01				0.04**	0.02**				0.03	0.00
Constant	2.97	0.03				2.81	0.03				2.05	0.03			
Frequency of contact	–0.07	0.04	–0.13			0.03	0.04	0.05			0.01	0.04	0.01		
Contact quality	0.10	0.04	0.18*			–0.10	0.04	−0.18*			–0.10	0.04	−0.18*		
Health status of grandparents	0.01	0.04	0.03			–0.04	0.03	–0.07			0.00	0.03	–0.00		
Contact quality × Health status of grandparents	0.05	0.03	0.10			–0.07	0.03	−0.14*			–0.01	0.03	–0.03		
Model 5				0.08**	0.05**				0.07**	0.03				0.09**	0.06**
Constant	2.26	0.43				2.92	0.42				2.61	0.43			
Frequency of contact	–0.07	0.04	–0.13			0.02	0.42	0.04			0.00	0.04	0.00		
Contact quality	0.10	0.04	0.17*			–0.10	0.04	−0.17*			–0.10	0.04	−0.16*		
Health status of grandparents	0.00	0.04	0.01			–0.03	0.04	–0.05			0.01	0.03	0.02		
Contact quality × Health status of grandparents	0.06	0.03	0.12*			–0.07	0.03	−0.15*			–0.02	0.03	–0.04		
Age	0.02	0.01	0.10			–0.01	0.01	–0.04			–0.02	0.01	–0.07		
Sex	0.01	0.08	0.01			0.12	0.07	0.10			0.10	0.07	0.08		
Contact to older people outside the family	–0.05	0.10	–0.03			0.06	0.10	0.04			0.01	0.10	0.01		
Personal health status	0.16	0.04	0.21**			–0.10	0.04	−0.13*			–0.15	0.04	−0.21**		

*p < 0.05, **p < 0.001.

**TABLE 4:** The role of grandparents for young adults’ age stereotypes (AS).

	Continued growth (CG_AS)					Physical decline (PD_AS)					Social loss (SL_AS)				
Variables	*B*	*SE* (*B*)	β	*R* ^2^	*ΔR* ^2^	*B*	*SE* (*B*)	β	*R* ^2^	*ΔR* ^2^	*B*	*SE* (*B*)	β	*R* ^2^	*ΔR* ^2^
**Model 1**															
Constant	2.40	0.04		0.00	0.00	3.22	0.03		0.00	0.00	2.60	0.03		0.00	0.00
Frequency of contact	–0.02	0.04	–0.03			–0.01	0.03	–0.01			–0.01	0.04	–0.01		
**Model 2**															
Constant	2.40	0.04		0.00	0.00	3.22	0.03		0.01	0.01	2.60	0.03		0.01	0.01
Frequency of contact	–0.01	0.04	–0.01			0.02	0.04	0.04			0.03	0.04	0.06		
Contact quality	–0.02	0.04	–0.04			–0.05	0.04	–0.08			–0.07	0.04	–0.13		
**Model 3**															
Constant	2.40	0.04		0.00	0.00	3.22	0.03		0.01	0.00	2.60	0.04		0.01	0.00
Frequency of contact	–0.00	0.04	–0.00			0.01	0.04	0.02			0.03	0.04	0.05		
Contact quality	–0.02	0.04	–0.04			–0.05	0.04	–0.09			–0.07	0.04	–0.13		
Health status of grandparents	–0.02	0.04	–0.04			0.03	0.03	0.05			0.03	0.04	0.05		
**Model 4**															
Constant	2.40	0.04		0.01	0.01	3.22	0.03		0.01	0.00	2.60	0.46		0.01	0.00
Frequency of contact	–0.00	0.04	–0.00			0.01	0.04	0.02			0.03	0.04	0.05		
Contact quality	–0.02	0.04	–0.03			–0.05	0.04	–0.10			–0.08	0.04	–0.13		
Health status of grandparents	–0.02	0.04	–0.03			0.02	0.03	0.04			0.03	0.04	0.05		
Contact quality × Health status of grandparents	0.02	0.03	0.04			–0.04	0.03	–0.08			–0.01	0.03	0.02		
**Model 5**															
Constant	2.63	0.48		0.03	0.02	2.85	0.43		0.03	0.02	2.40	0.46		0.04	0.03
Frequency of contact	–0.00	0.04	–0.00			0.01	0.04	0.01			0.02	0.04	0.04		
Contact quality	–0.03	0.04	–0.05			–0.04	0.04	–0.08			–0.06	0.04	–0.11		
Health status of grandparents	–0.04	0.04	–0.06			0.03	0.03	0.05			0.04	0.04	0.08		
Contact quality × Health status of grandparents	0.02	0.03	0.03			–0.04	0.03	–0.07			–0.01	0.03	–0.02		
Age	–0.02	0.01	–0.06			0.00	0.01	0.01			0.01	0.01	0.03		
Sex	–0.02	0.08	–0.01			0.13	0.07	0.11			0.13	0.08	0.10		
Contact to older people outside the family	–0.23	0.10	−0.13*			0.09	0.09	0.06			0.14	0.10	0.09		
Personal health status	0.08	0.05	0.10			–0.01	0.04	–0.02			–0.08	0.05	–0.10		

*p < 0.05.

**FIGURE 1:**
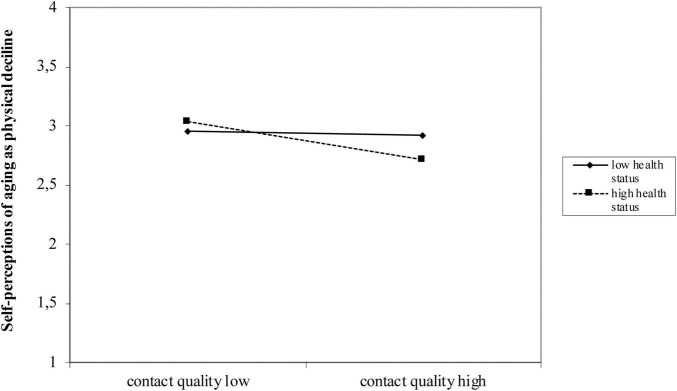
Moderation effect of grandparents’ health status on the relationship between contact quality and young adults’ self-perceptions of aging as physical decline.

To test hypothesis 3, which investigated the role of grandparents’ health, we also added grandparents’ health status to the models and found no significant effect for all three domains of VoA (Table 3, Model 3 for self-perceptions of aging, Table 4, Model 3 for age stereotypes). In addition, the effect of contact quality on self-perceptions of aging remained stable when including health status. In a next step, we introduced the moderator term, which showed a significant moderation effect of the grandparents’ health status on the relationship between contact quality and self-perception of aging as physical decline, Δ*R*^2^ = 4.17%, *F*(1, 285) = 5.6678, *p* < 0.019, 95% Cl [−0.2462, −0.0647]. Figure 1 shows the interaction effect between contact quality and grandparents’ health status. Higher contact quality was related to less self-perceptions of aging as physical decline (as indicated by lower scores on this scale), but only if grandparents’ health status is perceived as high. If grandparents’ health status is rated as low, high contact quality was not related to reduced negative self-perceptions of aging as physical decline.

**FIGURE 2:**
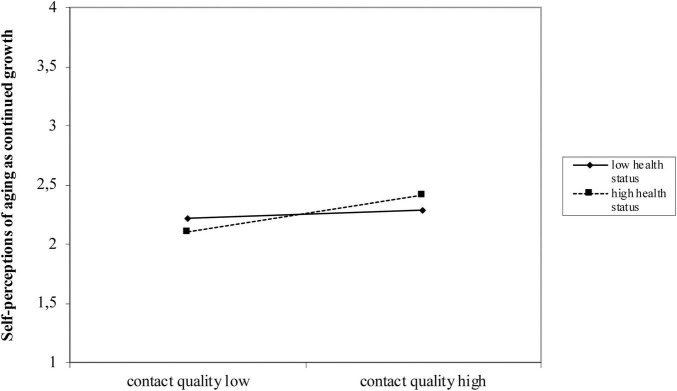
Moderation effect of grandparents’ health status on the relationship between contact quality and young adults’ self-perceptions of aging as continued growth.

When including age, gender, contact with older people outside the family and personal health status in a final step, all reported effects remained unchanged, except for the model predicting self-perceptions of aging as continued growth, for which the interaction effect became significant and mirrored that of physical decline (Table 3, Model 5). Figure 2 shows the interaction effect: The better the grandparents’ health, the more positive the self-perceptions of aging as continued growth, when the contact quality is higher. If the health status of the grandparents’ is poor, self-perceptions of aging as continued growth is independent of contact quality.

With regard to the effects of the control variables, personal health status of young adults was significantly related to self-perceptions of aging as continued growth, β = 0.21, *p* < 0.001 and negatively related to self-perceptions of aging as social loss, β = −0.21, *p* < 0.001 and physical decline, β = −0.13, *p* < 0.034 (Table 3, Model 5). In addition, contact with older people outside the family was significantly negatively related to age stereotypes as continued growth: Participants with contact had more positive VoA, β = −0.13, *p* < 0.026 (Table 4, Model 5).

### The Role of Family Factors

To address the relationship of young adults’ VoA with their parents’ VoA, as well as the frequency and valence of family conversations regarding age (family climate), we ran stepwise multiple regressions with the respective VoA as the outcome variable (see Tables 5, 6). Hypothesis 4, in which we assumed a positive relationship between parents’ and children’s VoA, was supported: Parents’ VoA were significantly related with their children’s’ VoA (Table 5, Model 1 for self-perceptions of aging; Table 6, Model 1 for age stereotypes). In terms of the fifth hypothesis, in which a positive relationship between VoA and family age climate was assumed, the frequency of talking about age in the family was neither related to the three domains of self-perceptions of aging (Table 5, Model 2) nor to age stereotypes (Table 6, Model 2). The valence of age-related family climate as an additional predictor, however, was significantly related to all three domains of self-perceptions of aging (Table 5, Model 2) but not for age stereotypes (Table 6, Model 2). When adding age, gender, health status, contact with older people outside the family, and parents’ age, the effect of parents’ VoA remained stable, whereas the effect of valence of family age climate remained significant only for self-perceptions of aging as physical decline. As for the effects of covariates, a small but significant negative effect of participants’ age was observed for self-perceptions of aging as continued growth, and personal health status was negatively related to self-perceptions of aging as social loss β = −0.13, *p* < 0.023 (Model 3, Table 5).

**TABLE 5:** The role of family factors for young adults’ self-perceptions of aging (SPA).

	Continued growth (CG_SPA)					Physical decline (PD_SPA)					Social loss (SL_SPA)				
Variables	*B*	*SE (B)*	β	*R* ^2^	*ΔR* ^2^	*B*	*SE* (*B*)	β	*R*_2_	*ΔR* ^2^	*B*	*SE* (*B*)	β	*R* ^2^	*ΔR* ^2^
**Model 1**															
Constant	1.78	0.17		0.16**	0.16**	1.32	0.16		0.24**	0.24**	1.11	0.12		0.20**	0.20**
Parent’s views on aging	0.43	0.06	0.40**			0.50	0.06	0.49**			0.43	0.05	0.45**		
**Model 2**															
Constant	1.85	0.22		0.18**	0.02	1.94	0.24		0.30**	0.06**	1.63	0.20		0.23**	0.03**
Parent’s views on aging	0.38	0.07	0.36**			0.43	0.06	0.42**			0.40	0.05	0.41**		
Family age climate (frequency)	–0.06	0.05	–0.08			0.02	0.04	0.03			–0.05	0.04	–0.06		
Family age climate (valence)	0.09	0.04	0.13*			–0.17	0.04	−0.25**			–0.11	0.04	−0.16*		
**Model 3**															
Constant	1.45	0.53		0.22**	0.04	1.98	0.50		0.31**	0.01	1.88	0.53		0.26**	0.03
Parents’ views on aging	0.38	0.07	0.36**			0.43	0.06	0.43**			0.37	0.06	0.39**		
Family age climate (frequency)	–0.08	0.05	–0.10			0.02	0.04	0.03			–0.05	0.04	–0.06		
Family age climate (valence)	0.07	0.04	0.11			–0.15	0.04	−0.22**			–0.08	0.04	–0.11		
Age father	–0.01	0.01	–0.07			–0.01	0.01	–0.08			0.00	0.01	–0.03		
Age mother	0.02	0.01	0.09			0.00	0.01	0.04			0.00	0.01	0.03		
Age	0.03	0.01	0.14*			0.00	0.01	–0.01			0.00	0.01	–0.10		
Sex	–0.06	0.07	–0.04			0.09	0.07	0.07			0.09	0.07	0.08		
Contact to older people outside the family	–0.06	0.09	–0.03			0.03	0.09	0.02			0.02	0.09	0.01		
Personal health status	0.07	0.05	0.09			–0.03	0.04	–0.05			–0.10	0.04	−0.13*		

*p < 0.05, **p < 0.001.

**TABLE 6:** The role of family factors for young adults’ age stereotypes (AS).

	Continued growth (CG_AS)					Physical decline (PD_AS)					Social loss (SL_AS)				
Variables	*B*	*SE* (*B*)	β	*R* ^2^	*ΔR* ^2^	*B*	*SE (B)*	β	*R* ^2^	*ΔR* ^2^	*B*	*SE* (*B*)	β	*R* ^2^	*ΔR* ^2^
**Model 1**															
Constant	1.50	0.20		0.07**	0.07**	2.00	0.16		0.19**	0.19**	1.79	0.13		0.14**	0.14**
Parent’s views on aging	0.31	0.07	0.27**			0.42	0.05	0.44**			0.38	0.06	0.37**		
**Model 2**															
Constant	1.29	0.25		0.08**	0.01	2.24	0.24		0.20**	0.01	1.85	0.23		0.14**	0.00
Parent’s views on aging	0.28	0.07	0.25**			0.41	0.06	0.43**			0.40	0.06	0.38**		
Family age climate (frequency)	0.05	0.05	0.06			–0.03	0.04	–0.04			–0.06	0.05	–0.06		
Family age climate (valence)	0.05	0.05	0.06			–0.04	0.04	–0.06			0.03	0.04	0.03		
**Model 3**															
Constant	2.09	0.57		0.10**	0.02	1.66	0.48		0.21**	0.02	0.61	0.61		0.17**	0.03
Parents’ views on aging	0.29	0.07	0.25**			0.41	0.06	0.43**			0.41	0.06	0.40**		
Family age climate (frequency)	0.05	0.05	0.06			–0.03	0.04	–0.04			–0.07	0.05	–0.08		
Family age climate (valence)	0.05	0.05	0.06			–0.03	0.04	–0.05			0.05	0.05	0.06		
Age father	–0.01	0.06	–0.07			–0.01	0.01	–0.06			0.00	0.01	–0.04		
Age mother	0.00	0.08	0.00			0.01	0.01	0.09			0.01	0.01	0.10		
Age	0.00	0.02	–0.01			0.00	0.01	–0.02			0.01	0.01	0.05		
Sex	–0.02	0.08	–0.02			0.06	0.07	0.06			0.13	0.08	0.10		
Contact to older people outside the family	–0.19	0.12	–0.11			0.07	0.09	0.05			0.18	0.10	0.11		
Personal health status	–0.03	0.05	–0.03			0.04	0.04	0.05			0.01	0.05	0.01		

*p < 0.05, **p < 0.001.

## Discussion

Perceptions of aging and older people develop early in life and have an influence throughout the life span, by impacting how younger people behave toward older people and also by becoming templates for one’s own aging process, ultimately influencing health and wellbeing in later life (Levy, 2009). Intergenerational contact with grandparents and the family as a context of attitude and value development in general are so far not well understood. The current study thus set out to investigate family factors related to young adults’ VoA in order to better understand the contexts influencing VoA development in early life.

### The Role of Grandparents: Intergenerational Contact Within the Family

Referring to studies showing the important role of contact with grandparents for VoA development (Christian et al., 2014; Gualano et al., 2018; Flamion et al., 2020b), we set out to investigate how contact frequency and quality as well as grandparents’ health status are related to young adults’ VoA in different dimensions (continued growth, physical decline, and social loss). We found that contact quality was significantly related to all domains of self-perceptions of aging (supporting Hypothesis 2), whereas for contact frequency no significant associations were observed (not supporting Hypothesis 1). Having a warm and meaningful positive relationship with one’s grandparents thus seems to be important for how one thinks about one’s own age.

However, as expected, grandparents’ health status emerged as a significant moderator between contact quality and self-perceptions of aging as physical decline (supporting Hypothesis 3): When contact quality was high, participants who perceived their grandparents’ health status as good had less negative self-perceptions of aging as physical decline. If grandparents’ health was rated as bad, however, high contact quality was not related to reduced negative self-perceptions of aging as physical decline. Therefore, having a sick or frail grandparent seems to override the positive effect of contact quality on young adults’ views of their own aging in terms of health. Grandparents’ negative health status thus seems to strengthen the negative view of one’s own aging as physical decline, which cannot be improved by high quality contact. However, not only for self-perceptions of aging as physical decline but also for self-perceptions as continued growth, a moderation effect could be observed after the inclusion of covariates. Again, for those with high contact quality who also perceived their grandparents’ health as good, self-perceptions of aging as continued growth were higher. If health status of grandparents was estimated as low, high contact quality was not related to improved self-perceptions of aging as continued growth. This knowledge is important, since young adults with sick grandparents might especially benefit from multifaceted knowledge on aging as well as from contact with older people outside the family to diversify their picture of aging. Further research could investigate whether similar moderators exist for other domain specific VoA of young adults, for instance social inclusion of grandparents in relation to social losses.

The majority of our participants (*n* = 265, 87%) had no contact with older people outside their family. This result matches those of Drury et al. (2016) and Newsham et al. (2021), who showed that intergenerational contact outside the family is rare and that grandparents are often the only older people that young adults have contact with. In addition, in our study, having contact with older people outside the family was only a relevant predictor for age stereotypes as continued growth. This stresses the importance of grandparents for VoA development, and the need to understand the conditions under which this contact has a positive effect. However, grandparents are usually seen as different exemplars compared to older people in general. Newsham et al. (2021) investigated college students’ perceptions of older people compared to those of their grandparents and found that participants attributed more negative emotional terms to older people outside the family than to their grandparents. Again, this calls for more diverse and also meaningful contact with older people outside the family, to enrich age stereotypes with different examples and to make young people aware of the diversity of the aging process.

### The Role of Family Factors: Family as a Context of Views on Aging Development

In accordance with the assumption that family serves as an important context of VoA development, similarities were observed regarding young adults’ VoA and those of their parents, supporting our fourth hypothesis. These associations remained stable even when controlling for age, gender, contact with older people outside the family and the personal health status of young adults. In terms of hypothesis 5 in which we tested whether family age climate was related to VoA, the frequency of talking about age in the family was not related at all to young adults’ VoA. The valence of age-related family climate was positively associated with all three domains of self-perceptions of aging. However, when adding the control variables, the effect of valence of family age climate was maintained only for self-perceptions of aging as physical decline. It seems that frequently talking about age without focusing specifically on positive aspects of aging, might not be enough to perpetuate more positive VoA, maybe because the discussion of more negative or rather mixed content rather confirms or cements prevailing negative views.

The results concerning the role of family aspects as a context of VoA development are in line with previous research on the transmission of values, attitudes, and other constructs such as for example gender roles, risk, and race attitudes within the family (Albert and Ferring, 2012; Gauly, 2017; Bredtmann et al., 2020; Wolff, 2020), and also with the musings of Stereotype Embodiment Theory, which stresses the importance of early age stereotype socialization for development across the life span (Levy, 2009). Further research should be specifically aimed at identifying the role of parents in the development of young adults’ VoA to get a better understanding of how parents’ perceptions of aging could have an influence on the VoA of young adults, similar to what has recently been done with older adults and their partners’ VoA (e.g., Kim et al., 2018). It seems that a positive age climate within the family is a powerful resource to shape young persons’ views of their own aging. This knowledge might be helpful in designing interventions to improve and diversify VoA which target the parents’ VoA or even those of the whole family system, for example by motivating resource-focused conversations about aging and older people (for example discussing newspaper articles or movies) and implementing them in families’ daily lives.

However, similarity within families in terms of values and attitudes is not only a matter of value transmission, but also of genetic similarities (Hufer et al., 2020). With regard to VoA, Kornadt and Kandler (2017) found in a sample of older twins that variability in multidimensional age stereotypes was due to both genetic and environmental influences. It would be interesting to consider these aspects in young adults in order to understand the role of family factors beyond shared environmental experiences and to better understand gene-environment interplay and their developmental relevance in sophisticated research designs (Mönkediek et al., 2020).

### The Role of Multidimensionality

Given that VoA are multidimensional in nature (e.g., Kornadt and Rothermund, 2011), and that this multidimensionality is already visible early in life (Kornadt et al., 2020), our study adds to previous research by focusing not only on VoA in different functional domains (continued growth, physical decline and social losses), but also young adults’ self-perceptions of aging as well as their views of older people in general, i.e., their age stereotypes. Both constructs are related, but also distinct, as is visible by their medium-sized correlations. Overall, relationships were strongest for self-perceptions, both for grandparents and family factors. Grandparents seem to be less important for young adults’ age stereotypes, this might be due to the previously mentioned fact that grandparents are exemplars that are not generalized to the older population in general (Newsham et al., 2021). In addition, seeing a family member age might have special relevance for the anticipation of one’s own aging and less for that of other people. With regard to the role of family age climate, effects were robust only for VoA as physical decline. Talking positively about aging within the family might thus combat negative instead of promote positive VoA. This shows the importance of a multidimensional assessment, since different facets of VoA might develop as a result of different factors, and thus only their consideration enables a holistic understanding (Kornadt and Rothermund, 2015; Kornadt et al., 2020).

### Limitations and Directions for Future Research

Some limitations have to be taken into account when interpreting our results, that point to directions for future research. First, it should be noted that the implications we draw from our analysis are limited by the cross-sectional nature of our data and therefore no directional relationships or developmental processes could be investigated. Thus, longitudinal research with samples at different ages is needed that is able to follow up on the development of young people’s VoA over time, and to investigate at which times in life family factors are most important and influential. Another significant limitation is that grandparents’ health status as well as parents’ VoA were obtained by proxy report from participants, which might have biased the results and resulted in inflated correlations. However, correlations were of medium size and the size varied for the different VoA indicators so there was by no means a complete match (or complete distancing) in terms of young adults’ report of their parents’ VoA. Nevertheless, truly dyadic or triadic data is absolutely necessary to confirm the results of our study. Furthermore, our sample was mainly composed of students (59%), in particular psychology students from the University of Luxembourg. Thus, the sample is rather homogeneous and precludes the generalization of effects to more diverse or even the general population. To reach more young people with different educational and cultural backgrounds, multilingual questionnaires could have been proposed, taking into account the diversity of the Luxembourgish population. In addition, the unequal distribution of female and male participants has a limiting effect on the generalizability of the results.

Some other limitations are related to the operationalization of the variables. In our survey, contact as well as the health status of the grandparents was not collected specifically for each grandparent but in general. In case of a discrepancy regarding the contact or the health status, participants were instructed to refer to the grandparent with whom they have the most contact. This might not correspond to the real situation for all grandparents. To obtain a more specific image of the situation, variables for each grandparent should be recorded. Furthermore, we did not assess whether our participants or their parents acted as caregivers. Having a grandparent or other family member in need of care might have an especially large impact on VoA and should therefore be included in further research. Finally, our study was focused on family-related factors that impact young adults’ VoA, however, family contexts become less important in young adulthood, thus, it would be interesting to investigate, other aspects which could influence young adults’ VoA development, for example teachers’ or peers’ VoA (Lineweaver et al., 2017) and also the age climate that is conveyed in classrooms or peer interactions.

## Conclusion

The results of this study reinforce the assumption that intergenerational contact within the family as well as different family factors, such as family climate and parents’ VoA, represent important aspects which could have an influence on the domain specific VoA of young adults. Given the importance of intergenerational dialogue when it comes to future developmental challenges, be it climate change or the current COVID-19 pandemic, understanding how VoA develop and which factors may be relevant for promoting a multifaceted, appreciative view of older age and older people, might facilitate “aging well” not only for individuals, but also for society as a whole.

## Data Availability Statement

The datasets presented in this study can be found in online repositories. The names of the repository/repositories and accession number(s) can be found below: https://osf.io/z74vf/?view_only=090e504449c7435b8769db6721ccbe14.

## Ethics Statement

The study received ethics approval under the regulations set by the Ethics Review Panel of the University of Luxembourg for final year theses. The patients/participants provided their written informed consent to participate in this study.

## Author Contributions

CH conceptualized the study, developed the questionnaire, carried out the data collection, and performed the analyses under the supervision of AK. CH wrote the first draft of the manuscript. AK commented and revised the manuscript. Both authors contributed to the article and approved the submitted version.

## Conflict of Interest

The authors declare that the research was conducted in the absence of any commercial or financial relationships that could be construed as a potential conflict of interest.

## Publisher’s Note

All claims expressed in this article are solely those of the authors and do not necessarily represent those of their affiliated organizations, or those of the publisher, the editors and the reviewers. Any product that may be evaluated in this article, or claim that may be made by its manufacturer, is not guaranteed or endorsed by the publisher.

## Footnotes

1 Adding the interaction between contact frequency and contact quality did not yield any significant additional effect (all *B* < 0.05, all *p* > 0.08).
